# State-of-the-Art Review on Amorphous Carbon Nanotubes: Synthesis, Structure, and Application

**DOI:** 10.3390/ijms242417239

**Published:** 2023-12-07

**Authors:** Xiaona Ren, Muhammad Irfan Hussain, Yue Chang, Changchun Ge

**Affiliations:** School of Materials Science and Engineering, University of Science and Technology Beijing, Beijing 100083, China; irfanustb@163.com (M.I.H.); changyue@ustb.edu.cn (Y.C.); ccge@mater.ustb.edu.cn (C.G.)

**Keywords:** amorphous carbon nanotubes, supercapacitor, lithium-ion battery, microwave absorption, nanohybrid composites

## Abstract

Carbon nanotubes (CNTs) have rapidly received increasing attention and great interest as potential materials for energy storage and catalyst fields, which is due to their unique physicochemical and electrical properties. With continuous improvements in fabrication routes, CNTs have been modified with various types of materials, opening up new perspectives for research and state-of-the-art technologies. Amorphous CNTs (aCNTs) are carbon nanostructures that are distinctively different from their well-ordered counterparts, such as single-walled and multi-walled carbon nanotubes (SWCNTs and MWCNTs, respectively), while the atoms in aCNTs are grouped in a disordered, crystalline/non-crystalline manner. Owing to their unique structure and properties, aCNTs are attractive for energy storage, catalysis, and aerospace applications. In this review, we provide an overview of the synthetic routes of aCNTs, which include chemical vapor deposition, catalytic pyrolysis, and arc discharge. Detailed morphologies of aCNTs and the systematic elucidation of tunable properties are also summarized. Finally, we discuss the future perspectives as well as associated challenges of aCNTs. With this review, we aim to encourage further research for the widespread use of aCNTs in industry.

## 1. Introduction

Amorphous carbon nanotubes (aCNTs) are a class of carbon-based nanostructured materials which are widely used in various fields like energy storage, electronics, catalysts, biomedical applications, and environmental industry [1,2,3,4,5]. As is well known, the famous single-walled, double-walled, or multi-walled CNTs (SWCNTs, DWCNTs, or MWCNTs, respectively) are characterized by curly or entangled, and graphitic sidewalls. Moreover, the properties of SWCNTs are affected by their chirality, and SWCNTs with different chiral structures show different properties [6]. However, aCNTs have attracted considerable attention thanks to their peculiar features, such as their straight tubular shape, dangling bonds on sidewalls, wide interlayer space, and amorphous and graphitic composed sidewalls, which remain unaffected by chirality and ensure easy large-scale preparation [7,8,9,10]. These materials exhibit a well-defined interface mechanism via the formation of robust covalent bonds between *sp*^2^- and *sp*^3^-hybridized carbon atoms, among which *sp*^2^ atoms create graphitic structures in the form of hexagonal honeycomb-like patterns and *sp*^3^ atoms in a tetrahedral configuration produce three-dimensional (3D) diamond-like structures [11,12]. Benefiting from their unique structural and physicochemical properties, a dynamically tunable approach can be proposed to achieve high efficiency and to control performance through component variability [13].

Despite their high importance, CNTs have faced persistent challenges concerning precise control of their properties, the consumption of gas in their preparation, and production scalability at low costs [14,15]. In addition, their potential toxicity, bioaccumulation, and environmental stability require further extensive research. To overcome these issues, most studies have been focused on SWCNTs and MWCNTs consisting of cylindrical graphene sheets. Meanwhile, very few studies have been dedicated to aCNTs to highlight their straight tubular structures and economical benefits: according to the data from the Web of Science platform, topics considering “amorphous carbon nanotube” account for only 97 studies, while those on “carbon nanotubes” account for more than 135,000 studies.

Synthetic strategies play a pivotal role in the rational design and selective fabrication of aCNTs to tailoring their structure and ensure desired morphologies, controlled sizes, and preferred functionalities [16,17]. Several authors have harnessed the advantages of aCNT fabrication to explore new frontiers in nanotechnology and exciting avenues for investigation and technological innovation. Another advantage of aCNTs is their large surface area, which has tunable porosity and is attractive for capacitors and Li-ion batteries (LIBs) [18]. For instance, a record thermal conductivity of 0.075 W·m^−1^·K^−1^ (theoretical value) has been obtained for aCNTs, demonstrating their exceptional thermal stability and oxidation resistance below 300 °C; moreover, they could transform into MWCNTs at 1700 °C in argon atmosphere [19,20,21]. Furthermore, the diverse monolayer aCNTs exhibit intriguing optical characteristics and are thus promising candidates for various applications, including optoelectronics, sensors, and ultraviolet filters [22,23].

The scope of this review is to provide conceptual definitions and to report the latest progress in the engineering of aCNT materials, as well as their synthesis routes and potential application fields. In addition, we emphasize the importance of structural tunability for the production of highly efficient and stable carbon nanostructures.

## 2. Comparison of the CNTs

As shown in Table 1, the most obvious difference between aCNTs and conventional CNTs like SWCNTs, MWCNTs, or DWCNTs is their sidewall formed by amorphous carbon atoms, and this lead to features of aCNTs different with graphitic CNTs. The graphene lattice base vectors *a*_1_ and *i*_2_ lead to a critical parameter of CNT, which is a graphene layer rolled-up vector (*m*,*n*). The (*m*,*n*) indices determine diameter and chirality (*θ*, the chiral angle between hexagons and the tube axis) of a CNT [24]. Since SWCNTs are formed by one-layer graphene, their electronic properties depend on their chirality, while the other graphitic CNTs have more than one layer and the chirality effect is inconclusive.

**Figure 1:**
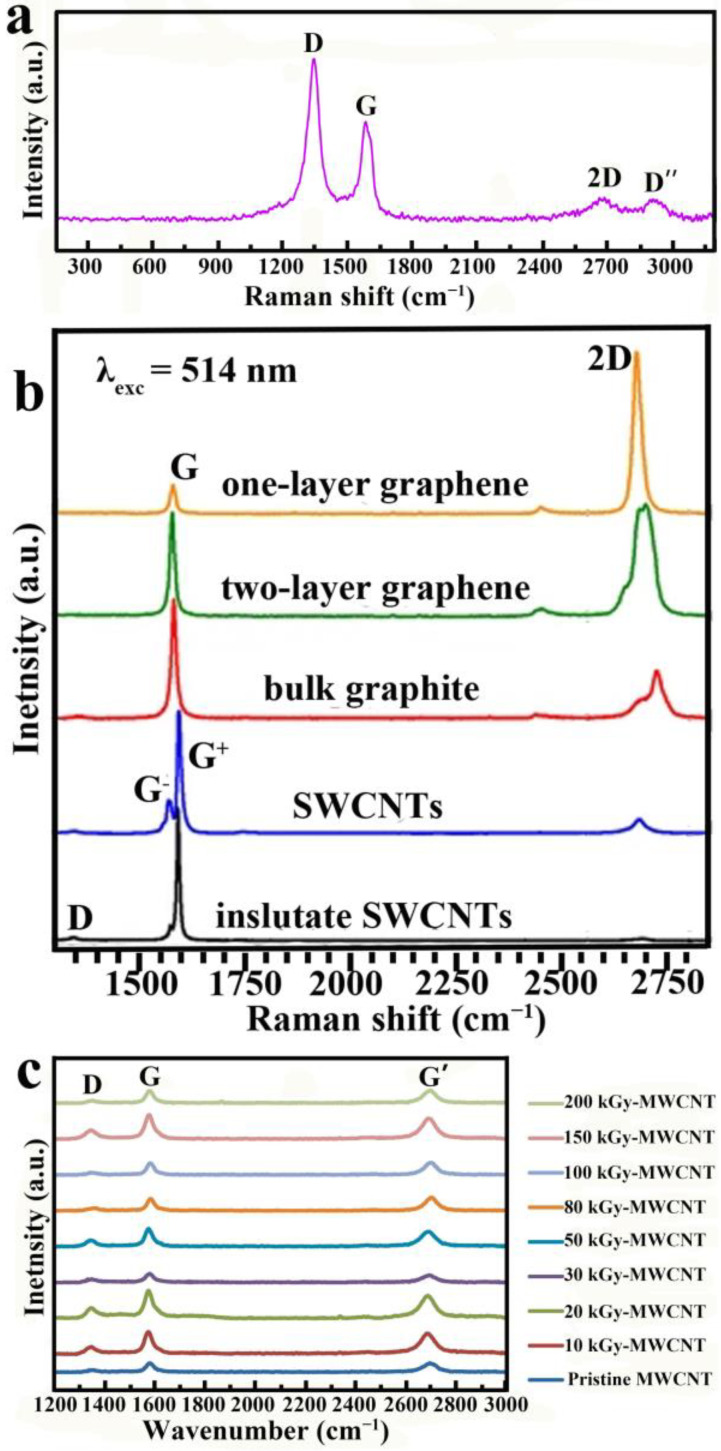
Typical Raman spectra of (**a**) aCNTs, adapted from Ref. [27], copyright 2017, Elsevier; (**b**) Raman spectra of graphene and SWCNTs, adapted from Ref. [28], copyright 2016, Springer; and (**c**) Raman spectra of MWCNTs with or without electron irradiation, adapted from Ref. [29], copyright 2023, Elsevier. D band is corresponding to amorphous carbon; G band is corresponding to graphite carbon; 2D, D’’ and G’ band are correspong to graphene carbon; G^+^ band is corresponding to valence vibrations of armchair nanotubes perpendicular to nanotube axis; G^−^ band is corresponding to valence vibrations of zigzag nanotubes parallel to the nanotube axis.

In order to ascertain the CNTs’ graphitized structure, Raman spectroscopy is one of the most helpful methods, in which the D-band at 1341 (or 1370) cm^−1^ and G-band at 1602 (or 1581 [25]) cm^−1^ correspond to amorphous and graphitic carbon, and their intensity ratio I_D_/I_G_ is the key parameter representing the crystallinity of CNTs [26]. For example, an I_D_/I_G_ ratio of 1.364 indicates more disordered carbon in the wall of aCNTs (Figure 1a) [27], while an I_D_/I_G_ ratio lower than 1 is always derived from graphitic CNTs, such as the I_D_/I_G_ ratio of SWCNTs being much lower than 1 (Figure 1b) [28], and the same as MWCNTs (Figure 1c), even after different intensity electron irradiations [29]. Particularly, the I_D_/I_G_ ratio of graphene is 0 (Figure 1b) [28] and the I_D_/I_G_ of MWCNTs is obviously higher than SWCNTs (Figure 1b,c), since MWCNTs have more defects than SWCNTs. On the other hand, due to the amorphous carbon sidewall, the functional properties of aCNTs, such as the ability to dissolve lithium, are inferior to those of graphitic CNTs [30]. This is the reason why aCNTs have not received as much attention as graphitic CNTs. However, the unique amorphous sidewall of aCNTs allows them to be allied as templates, e.g., in the controllable preparation of nanostructured tungsten or tungsten carbide [31].

**Table 1:** Comparison of various types of CNTs [6,7,8,9,10,32].

CNTs	Sidewall Carbon Atom	Sidewall Layers Number	Nanotube Shape	Defects Quantity	Property Dependence on Structural Defects
aCNTs	Amorphous and graphitic carbon atoms	Several	Straight tubular structure in most cases, coiled, brushes, “test tube”-like; one open end or both ends open; and coral-like aCNTs in a few cases	Plenty	Slightly
SWCNTs	Graphitic carbon atoms	One	Curly or entangled, bundles in most cases; sometimes can be serpentine, cross-bar, or with specific turning angles	Nearly none	Severe
DWCNTs	Graphitic carbon atoms	Two	Curly or entangled	Nearly none	Severe
MWCNTs	Graphitic carbon atoms	Several	Curly or entangled	Modicum	Slightly

**Figure 2:**
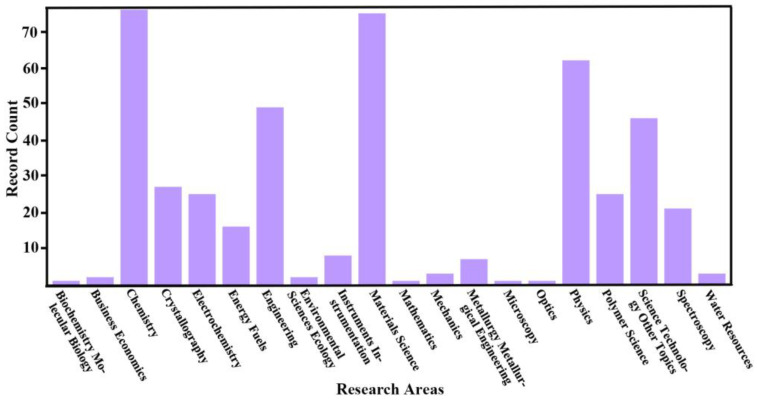
Analysis of the papers reporting amorphous carbon nanotube according to Web of Science database.

**Figure 3:**
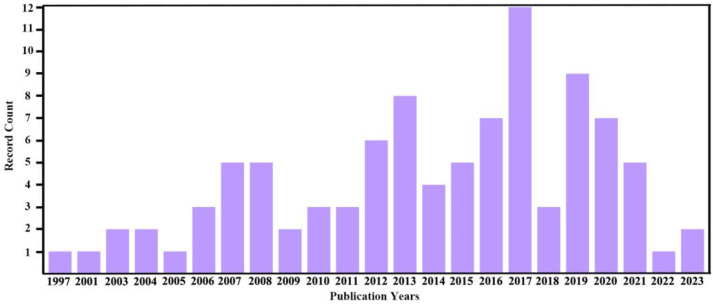
Publication years of the papers concerning amorphous carbon nanotube according to Web of Science database.

As aforementioned, there are more than 135,000 reports concerning carbon nanotubes, while the work on amorphous carbon nanotubes accounts for only 97. Among these 97 papers, the most employed research areas are chemistry and materials science (Figure 2), and most of the related publications date back to 2017 (Figure 3).

## 3. Synthesis Methods

The methods to fabricate aCNTs are the same as those for graphitic CNTs, including template techniques, chemical vapor deposition (CVD), catalytic pyrolysis, and arc discharge (see Table 2). However, unlike graphitic CNTs, aCNTs can be obtained on a large scale at temperatures even below 250 °C. The products of aCNT preparation can be pure aCNTs or aCNT nanohybrids. While a template is used as a tool based on the mechanism of aCNT formation, it is always combined with CVD or catalytic pyrolysis, which is similar to arc discharge, always requiring the presence of catalysts. The fabrication via catalytic pyrolysis can also be implemented at low temperatures or by generating a template during the process. Therefore, we summarize the preparation methods according to their typical characteristics.

**Table 2:** Typical preparation methods of aCNTs.

Method	Raw Materials	Product	Ref.
Template	V_3_O_7_·H_2_O as template and glucose as carbon source, via hydrothermal route	Hydrogenated aCNTs	[19]
	AAO	aCNTs composited with SnO_2_, MnO_2_/GO or bimetal oxide	[33,34,35,36]
	Sulfonated polymer nanotubes	aCNTs	[37]
	PVP as template and Ni as catalyst, via triple-coaxial electrospinning	aCNTs decorated with graphite nanospheres	[38]
	ZnO nanowires as template and formaldehyde resign as carbon source	aCNTs	[39]
	SnO_2_ nanowires as template and glucose as carbon source, via hydrothermal	aCNTs encapsulated with SnO_2_ nanowires	[40]
	Sulfonated polymer nanotubes	Thin-walled porous aCNTs	[41]
	PC membrane filters as template and glucose as carbon source	Amorphous carbon/PC membrane composite	[42]
	Aligned TiO_2_ nanotubes as template, via electrochemical deposition	Aligned aCNTs/TiO_2_	[43]
	Halloysite as template	aCNTs	[44]
CVD	Acetylene, Co/Ni-modified Si as catalyst	aCNTs encapsulated with Si	[27]
	Xylene, ferrocene, and triethylsilane	Network or aligned, coiled, V-shaped, and ribbon-like aCNTs with different raw material ratios	[45,46,47,48]
	Electron cyclotron resonance—CVD, AAM as template, acetylene and Ar as precursor, ca. 100 °C for 4 min	Aligned hydrogenated aCNTs	[49]
	Mesoporous silica SBA-15 as matrix, Fe_2_O_3_ as catalyst, and hexane as carbon source	aCNTs	[50]
	Co-Ni as catalyst and acetylene as carbon source	aCNTs	[51]
	Acetylene as carbon source and iron-coated indium tin oxide as substrate	Indium oxide encapsulated in aCNTs	[52]
Catalytic pyrolysis	Poly(tetrafluoroethylene) and ferrous chloride	aCNTs	[8,53]
	AAM as template and ferrocene as raw material	aCNTs encapsulated with iron oxide nanoparticles	[54,55]
	Fe-Co/CaCO_3_ as bimetallic catalyst and ethylene as carbon source	aCNTs or N-doped aCNTs (N_2_ as gas carrier)	[56,57,58]
	Co/RGO as catalyst and ethanol as carbon source	aCNTs/RGO composite	[59]
Low-temperature synthesis	Ferrocene and ammonium chloride	aCNTs	[9,60,61,62,63,64,65,66,67,68,69,70,71,72,73,74,75,76,77,78,79,80,81,82,83,84,85,86,87,88,89,90,91,92]
	Polycarbonate membrane as template, glucose decomposition	aCNT brushes	[25,93]
	Self-catalysis decomposition of Ni bis(dimethylglyoximate) at 250 °C	aCNTs or Ni-containing aCNTs	[94]
Self-catalysis decomposition	Ferrocene in benzene	Long aCNT bundles	[95]
Arc discharge	Co-Ni (1:1) alloy and graphite	aCNTs and soot	[96]
	Mo-Co_2_O_3_-Mg (1:1:8 wt.%) powders as catalyst and graphene sheets as carbon source		[97]
Thermal evaporation	Carbon paper as carbon source	aCNTs encapsulated with Sn	[98]

### 3.1. Template

**Figure 4:**
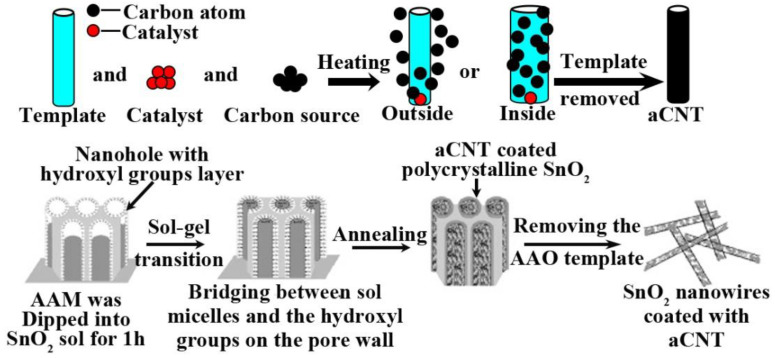
Illustration of the mechanism of the preparation of aCNTs via templates and an example of the production of aCNT-coated SnO_2_ nanowire. Adapted from Ref. [34], copyright 2010, Elsevier.

Amorphous CNTs can be obtained when templates such as nanowires or mesoporous materials (i.e., anodic alumina membrane (AAM)) are used, and carbon source materials are deposited or pyrolysis occurs on the nanowires or inside the mesopores (Figure 4). The diverse template methods to prepare aCNTs are summarized in Table 2. The common templates are V_3_O_7_·H_2_O [19], anodic alumina oxide membrane (AAOs) [33,34,35,36], sulfonated polymer nanotubes [37], polyvinyl pyrrolidone (PVP) [38], ZnO nanowires [39], SnO_2_ nanowires [40], sulfonated polymer nanotubes [41], polycarbonate (PC) membrane filters [42], aligned TiO_2_ nanotubes [43], and halloysite [44].

### 3.2. Chemical Vapor Deposition

**Figure 5:**
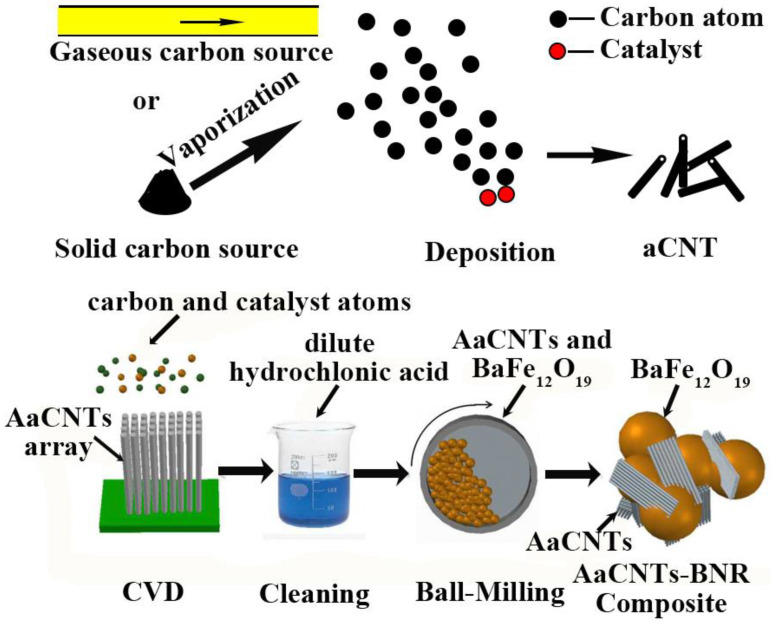
Illustration of the mechanism of the preparation aCNTs via CVD and an example of the production of aligned aCNT/BaFe_12_O_19_ (BF) (here, AaCNTs denote the aligned aCNTs). Adapted from Ref. [47], copyright 2017, Elsevier.

During the CVD process, a simple organic gas is the most used carbon source, and metal-containing catalysts are required for carbon source pyrolysis (Figure 5). Furthermore, a substrate or core is needed for the deposition of carbon atoms, so CVD is appropriate for preparing aCNT nanocomposites.

Networks of aCNTs on 3D graphene aerogel (GA)/BaFe_12_O_19_ (BF) as well as aligned aCNTs (AaCNTs), coiled aCNTs, V-shaped aCNTs, and ribbon-like aCNTs can be prepared via floating catalyst CVD whereby ferrocene and triethylsilane are used as catalyst precursors and carbon is derived from xylene [45,46,47,48,99]. Using electron cyclotron resonance CVD, hydrogenated aCNTs in an AAM template have been obtained from acetylene and argon at about 100 °C after 4 min, and the diameter of aCNT can be adjusted by changing the AAM diameter [49]. Applying the mesoporous silica SBA-15 as a matrix, Fe_2_O_3_ as catalyst, and hexane as a carbon source, aCNT was produced via CVD in [50]. By in situ CVD, aCNTs can be successfully deposited onto silicon particles to produce a 3D-structured Si/aCNTs composite [27].

### 3.3. Catalytic Pyrolysis

**Figure 6:**
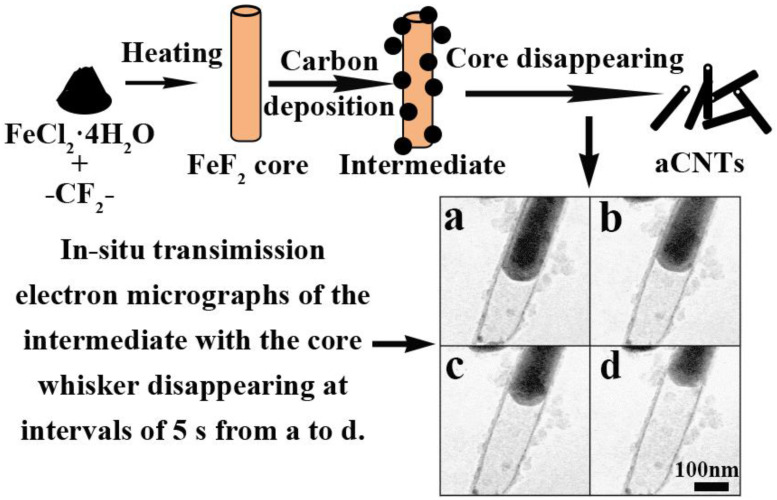
Illustration of the aCNT formation mechanism during the catalytic pyrolysis and in situ transmission electron micrographs of the core evolution at intervals of 5 s from a to d. Adapted from Ref. [53], copyright 2003, Elsevier.

Amorphous CNTs can be obtained by catalytic pyrolysis of poly(tetrafluoroethylene) and ferrous chloride (FeCl_2_) [8,53]. As confirmed by in situ TEM observations, this process generates templates, and aCNTs are formed in the following steps: core whiskers of a stable capsule-like ferrous fluoride form along one direction without any solid material supply below 550 °C; amorphous carbon deposits on the whiskers without any preferred orientation; the core whiskers then gradually vaporize above 650 °C, leaving aCNTs behind (Figure 6) [53].

In addition, self-catalytic decomposition of ferrocene in benzene at low temperatures (<210 °C) can result in long bundles of aCNTs [95]. Furthermore, confined by AAM, the pyrolysis of ferrocene yields the aCNTs encapsulated with the iron oxide [54]. Bimetallic Fe-Co/CaCO_3_-catalytic pyrolysis of ethylene or mixtures of ethylene and nitrogen is a method to prepare aCNTs or N-doped aCNTs [56,57]. Using typical triple-coaxial electrospinning, polyacrylonitrile (PAN, carbon source), nickel acetate (Ni(Ac)_2_, Ni catalyst source), and polyvinyl pyrrolidone (PVP, template) can be transformed into aCNTs decorated with nanospheres of graphitic carbon [38]. Furthermore, Co on reduced graphene oxide (Co/RGO) catalyzes the pyrolysis of ethanol to produce aCNTs/RGO nanocomposites [59].

### 3.4. Low-Temperature Synthesis

**Figure 7:**
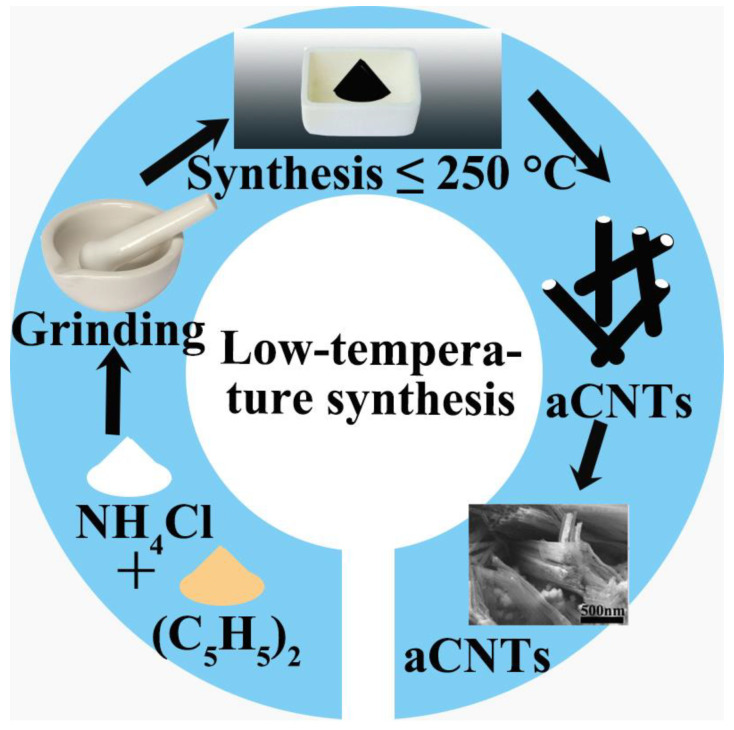
Scheme of aCNT preparation via low-temperature synthesis and the field emission scanning electron micrograph of as-obtained aCNTs. Adapted from Ref. [65], copyright 2012, Elsevier.

The typical low-temperature synthesis is conducted at temperatures lower than 250 °C and proceeds via the following steps (Figure 7): ferrocene ((C_5_H_5_)_2_Fe) and ammonium chloride (NH_4_Cl) with a weight ratio of 1:2 are ground in a mortar and then heated in an oven for 30 min at temperatures below 250 °C. After cooling, black aCNT powder is obtained. Due to simplicity of the process, the low synthesis temperature, and the large-scale production, this is the most common method to prepare aCNTs (Table 2).

In addition, by applying a polycarbonate membrane with an average particle diameter of 220 nm and a thickness in the range of 7–22 μm as a template, aCNT brushes can be released through the decomposition of glucose upon a hydrothermal process conducted at 180 °C for 6 h [25,93]. The open-ended aCNTs were also obtained via self-catalytic decomposition of Ni bis(dimethylglyoximate) at 250 °C [94].

### 3.5. Arc Discharge

**Figure 8:**
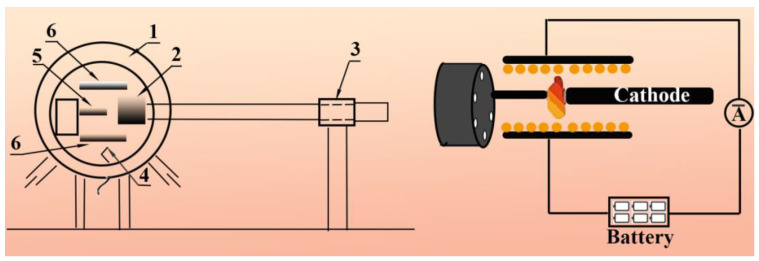
Schematic diagram of a modified arc discharge furnace, reproduced with permission from Ref. [101], copyright 2017, Springer: 1—vacuum chamber; 2—moving cathode; 3—feeding system of the electrode; 4—thermocouple; 5—turnable anode; 6—electrode plates.

Applying Co-Ni (1:1) alloy powder as a catalyst and graphite powders in the anode rods as a carbon source, aCNTs with diameters between 7 and 20 nm can be obtained via temperature-controlled arc discharge in a hydrogen atmosphere at 500 Torr (Figure 8) [96,100]. When using Mo/Co_2_O_3_/Mg (1:1:8 wt.%) powders as a catalyst at 600 °C, graphite sheets are transformed into coral-like aCNTs [97].

### 3.6. Others

In addition to the aforementioned preparation methods, aCNTs could be obtained via the solvothermal treatment of ferrocene and sulfur in a molar ratio of 1:2, while Fe/C coaxial nanocables can be produced at a ratio of 1:1 [102]. Through the 12 h hydrothermal treatment at 200 °C, aCNTs were generated from a toluene solution of ferrocenecarboxylic acid, carbon tetrachloride, and carbon disulfide [103]. The carbonization of a mixture of urea, boric acid, PEG-2000, and metal salts (CoCl_2_·6H_2_O, NiCl_2_·6H_2_O, and MnCl_2_) via metal cation-assisted pyrolysis produces a composite of amorphous metal encapsulated into amorphous B,N-co-doped CNTs [104].

Furthermore, ferrocene heated in a chlorine gas atmosphere for different reaction times at various temperatures can produce diverse nanocarbon products, e.g., aCNTs will be formed upon heating at 200 °C for 30 min [105]. Using a deposition–etching–evaporation technique and ZnO nanorods as a template, well-aligned aCNTs have been obtained [106]. Polymers growing freely on N-doped carbon surfaces at 600–800 °C in a nitrogen atmosphere can generate aCNTs or N-doped amorphous carbon [107].

**Figure 9:**
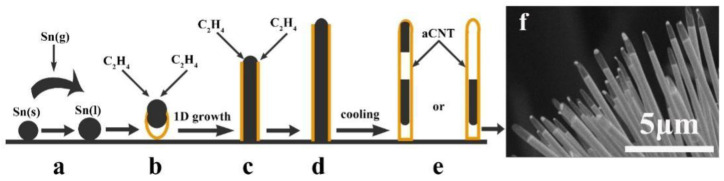
(**a**–**e**) Illustration of the growth mechanism of aCNT-Sn nanostructures: aCNT encapsulating single-crystalline Sn nanowires on a graphitic fiber. (**f**) Scanning electron micrograph showing the tip of the nanostructure array of aCNTs encapsulating the pure Sn nanowires. Adapted from Ref. [98], copyright 2007, ACS Publications. (**a**) Sn powder first vaporizes and then condenses to form nuclei of Sn droplets. (**b**) A CNT starts to grow at the bottom of the Sn droplet that acts as a catalyst, and the growth of 1D Sn nanowires is initiated by an autocatalytic VLS (vapor–liquid–solid) process. (**c**) The CNT and Sn nanowire continue to grow coaxially. (**d**) The CNT closes at the tip once the process is completed. (**e**) Cavities are formed within the nanowire or at the tip.

In addition, a pure Sn powder and a small piece of commercially available carbon paper were placed side by side in a furnace before heating to 900 °C for approximately 15 min in an argon flow containing 2% ethylene. After holding the temperature for 2 h and cooling to room temperature, aCNTs encapsulated with single-crystalline Sn nanowires have been obtained (Figure 9) [98].

## 4. Structure

**Figure 10:**
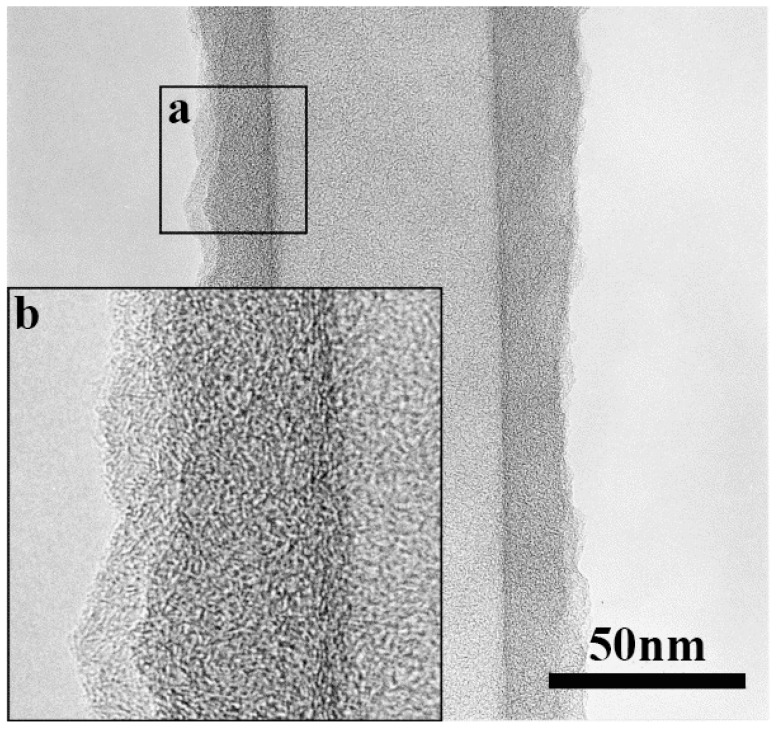
(**a**) Transmission electron micrographs of amorphous carbon wall of aCNT and (**b**) high magnification image of the enclosed region in image (**a**). Reproduced with permission from Ref. [8]. Copyright 2003, Elsevier.

**Figure 11:**
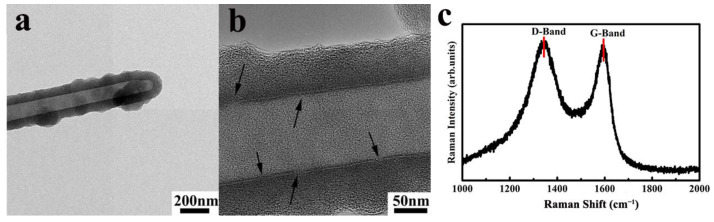
(**a**,**b**) Transmission electron micrographs of an aCNT sidewall, reproduced with permission from Ref. [110], arrows of (**b**) marks the discoutinuous inner wall of aCNTs, copyright 2016, Elsevier, and (**c**) Raman spectrum of aCNTs, reproduced with permission from Ref. [109], copyright 2019, IOP Science.

Amorphous CNTs consist of graphite and amorphous carbon in different ratios (Figure 10) and can be formed by rolling amorphous graphene [108]. The straight tubular structure is the most obvious characteristic of aCNTs that distinguishes them from graphitic CNTs. Other features such as the open end and the lack of chirality of aCNTs are also different from graphitic CNTs. As shown in Figure 10, the inner wall of aCNT is extremely straight and consists of aligned carbon atoms, while the outer surface appears in a zigzag pattern, consisting of randomly aligned carbon atoms [8]. Most interestingly, the authors have established that the inner wall of aCNT is discontinuous, as marked in Figure 11b, with an I_D_/I_G_ ratio of about 1.02 (Figure 11c) [109,110].

**Figure 12:**
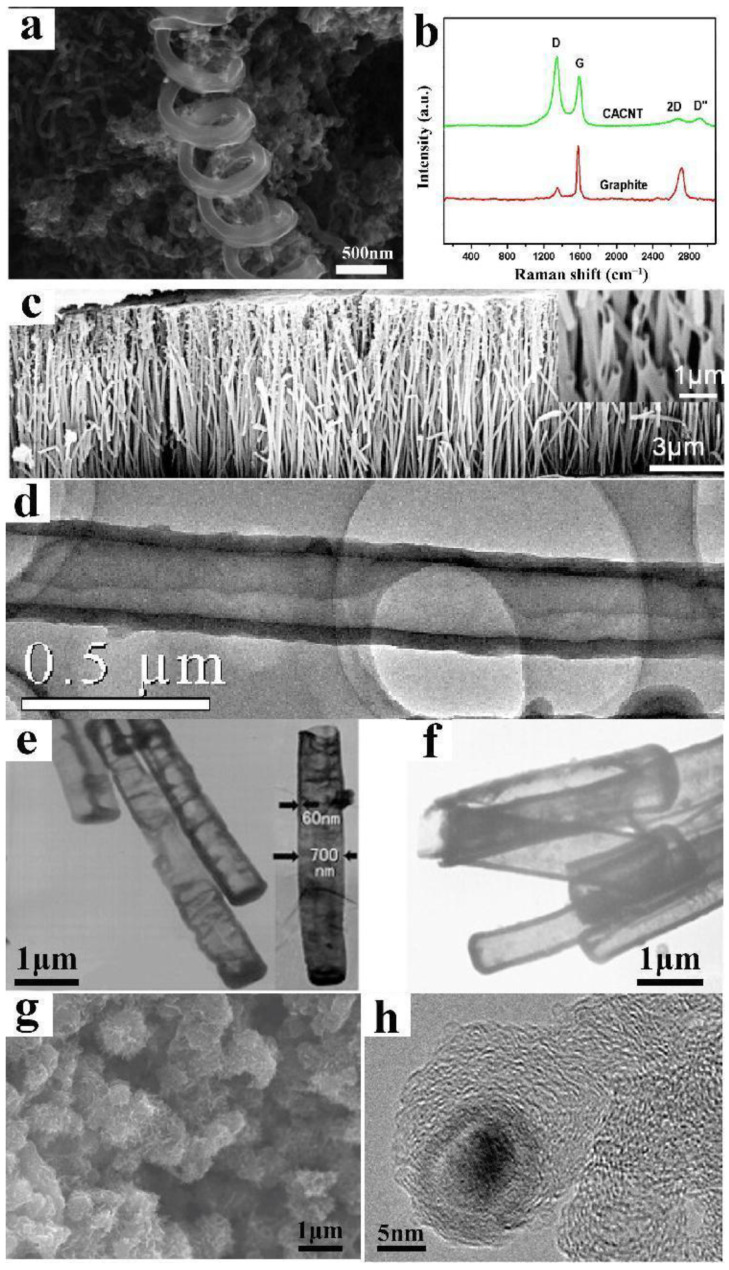
(**a**,**b**) Structure of CaCNTs, adapted from Ref. [99], copyright 2018, Elsevier; (**c**,**d**) aCNT brushes, adapted from Ref. [25], copyright 2007, ACS Publications; (**e**,**f**) “test tube”-like aCNTs, adapted from Ref. [94], copyright 2007, Elsevier; and (**g**,**h**) coral-like aCNTs, adapted from Ref. [97], copyright 2017, Taylor & Francis Online.

In addition to the straight tubular aCNT structure, there are coiled aCNTs (CaCNTs), aCNT brushes, “test tube”-like aCNTs with one open end, aCNTs with both ends open, and coral-like aCNTs (Figure 12) [25,94,97,99]. As shown in Figure 12b, the I_D_/I_G_ ratio of the CaCNT is greater than 1, showing a higher content of the amorphous carbon structure.

**Figure 13:**
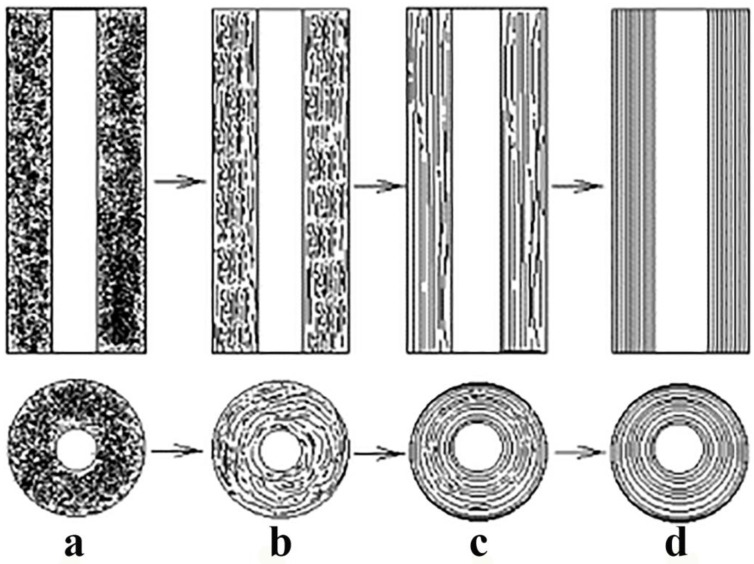
(**a**–**d)** Crystallization model of aCNTs with enlarged central cores, reproduced with permission from Ref. [111], copyright 2001, Elsevier.

**Figure 14:**
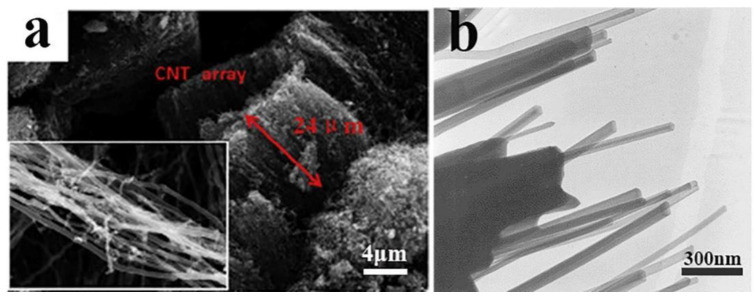
(**a**) Scanning electron micrograph, reproduced with permission from Ref. [47], copyright 2017, Elsevier, and (**b**) transmission electron micrograph of AaCNTs (AaCNTs: aligned aCNTs), reproduced with permission from Ref. [49], copyright 2000, Elsevier.

As aforementioned, aCNTs are thermally stable up to 300 °C. The amorphous carbon shell will crystallize at higher temperatures, i.e., the annealing at 1700–2200 °C in argon for 2 h results in the growth of graphitic crystallites and the formation of stiff and continuous turbostratic stack layers in aCNTs (Figure 13) [111]. Once the temperature is further increased, the typical non-graphitizable carbon changes to graphite below 2800 °C [112]. In most cases, aCNTs are disordered, but when the preparation process is controlled or a template is used, AaCNTs can be obtained (Figure 14) [47,49]. As the arrow shown in Figure 14a, we can see the length of AaCNTs is circa 24 μm, the inset of Figure 14a,b show the AaCNTs in detail.

## 5. Applications

Compared with SWCNTs or MWCNTs, aCNTs are easier to hybridize with other nanomaterials due to their unique structure characterized by the amorphous carbon shell and dense dangling bonds. Furthermore, the unique straight tube structure and open end endow aCNTs with a capillary adsorption ability. Additionally, thanks to sidewall defects, aCNTs have excellent reversible capacity and rate capacity [37]. This is because the properties of aCNTs not being affected by the chirality present in graphitic CNTs. However, the hydrophobicity of aCNTs, which is the same as that of graphitic CNTs, limits their application in aqueous solutions. Fortunately, similar to graphitic CNTs, the modification of aCNTs with oxygen-containing groups is beneficial to their application in aqueous media [110,113].

Remarkably, aCNTs have the potential to be used in various fields like dye or heavy metal removal, LIBs, field-emission displays, cold cathodes, electromagnetic wave absorbers, catalyst carriers, gas adsorbers, and templates (Table 3) [7,39,69,77,84,92]. Similar to amorphous materials, amorphous sidewalls endow aCNTs with the aforementioned features, while destroying the tubular nanostructure and impairing the properties of aCNTs compared to graphitic CNTs [37]. Therefore, the applications of aCNTs are more limited relative to SWCNTs or MWCNTs.

### 5.1. Electrical Applications

As aforementioned, aCNTs exhibit high specific capacities, which makes them promising candidates for application in energy storage devices such as supercapacitors and batteries. Remarkably, oxidized aCNTs can reach a capacity of 530 mAh/g, which remains at 93% even after five cycles [115]. The demonstrated high capacity of aCNTs is assumed to be due to the confinement and electrical conductivity of the aCNT channels as well as the defects and the large specific surface area, indicating the application potential of aCNTs in energy storage devices like LIBs [33,34,40,116,117], i.e., the aCNTs decorated with graphite nanospheres and those encapsulated with Sn are exceptional anode materials for high-power and high-energy LIBs [38,118]. Additionally, the aCNTs can enhance the cycling performance of SnO_2_ nanowire arrays, which possess high energy densities [33,34] Similarly, aCNT/RGO composites exhibit higher lithium storage properties than well-crystallized CNT-modified RGO electrodes [59]. Moreover, aCNTs are remarkable as part of Li-S batteries (LSBs) [41,51] and potassium-ion batteries (PIBs) [119]. Furthermore, when aCNTs are co-doped with B and N and encapsulated with amorphous metal, they can perfectly serve as sodium-ion battery anodes [104].

In addition, aCNT-based nanohybrids can be highly efficient supercapacitors. For instance, Si/aCNT structures can achieve a high capacity of 1496 mAh/g and a rate capacity of 808 mAh/g at a current of 100 mA/g, as well as superior cycling stability with 80% capacity retention after 300 cycles [27]. Other aCNTs decorated with NiO nanoflowers [76], MnWO_4_ nanorods [81], or MoS_2_ [120] exhibit outstanding supercapacitor potential with a specific capacitance of around 120 F/g (at a scan rate of 10 mV/s), 542.18 F/g (at a scan rate of 2 mV/s), and 511 F/g (at a scan rate of 5 mV/s), respectively. Furthermore, lithium hexafluorophosphate (LiPF_6_), ethylene carbonate (EC), and poly(ethylene oxide) (PEO) demonstrate improved conductivity when combined with aCNTs [121,122,123,124]. Finally, the dielectric properties of SiO_2_ networks can be increased to some extent when introducing aCNTs [60].

### 5.2. Absorption

Due to the wide interlamellar space, aCNTs are more versatile than graphitic CNTs in the field of absorption. In particular, composites of tubular aCNTs and other nanomaterials have excellent wave-absorbing abilities. Both nanocomposites of aCNTs deposited onto a 3D graphene aerogel (GA)/BaFe_12_O_19_ (BF) composite (aCNT/GA/BF) or composed with only BF showed good electromagnetic wave absorption performance, which is attributed to the multiple reflection and scattering of electromagnetic waves in aCNTs [44,46,47]. Similarly, the nanohybrid of aCNT-CdSe quantum dots shows great potential as an electromagnetic wave and microwave absorber and has good optical properties [9,86]. Combined with Fe_3_O_4_ or a 3D porous pyrolytic carbon (PyC) foam, aCNTs also exhibit potential for use as absorbers of electromagnetic waves [125,126]. In addition to the wide interlamellar space, the coiled structure has the advantage of a high reflection frequency inside the material, which makes CaCNTs promising as microwave adsorption materials. Compared with pristine CaCNTs, those decorated with La(NO_3_)_3_ and RGO, or co-modified with Fe, Co, and Ni show broader bandwidth, and the maximum absorption peak of CaCNTs can be effectively increased via modification with rare earth La^3+^ ions [99]. Furthermore, aCNTs can serve as water pollution adsorption materials in the removal of organic dyes [77] or rhodamine B from water [127] due to their abundant defects.

### 5.3. Supports and Templates

**Figure 15:**
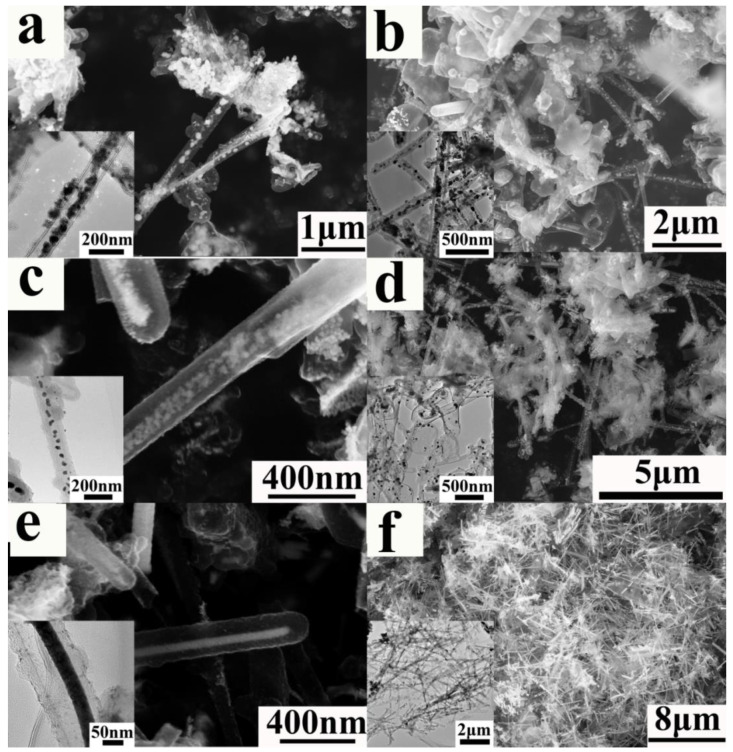
(**a**–**d**) Controllably prepared W nanodots with different densities and (**e**,**f**) nanowires utilizing aCNTs as template. The insets depict the related transmission electron micrographs, reproduced with permission from Ref. [31], copyright 2017, IOPscience.

**Figure 16:**
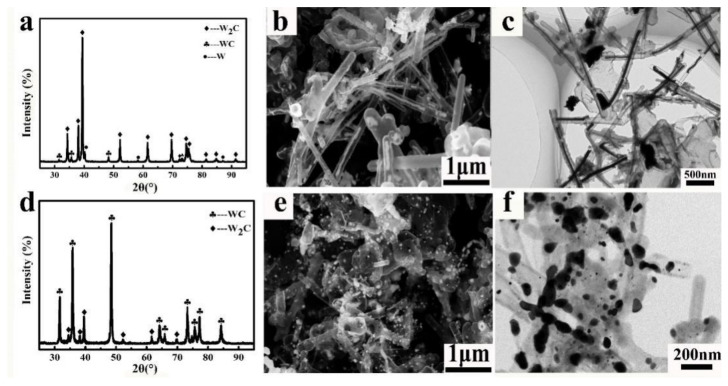
(**a**–**c**) Controllably prepared tungsten carbide nanowires and (**d**–**f**) nanodots with aCNTs as template, (**a**,**d**) XRD patterns; (**b**,**e**) field emission scanning electron micrographs; (**c**,**f**) transmission electron micrographs, adapted from Ref. [128], copyright 2017, IOPscience.

**Figure 17:**
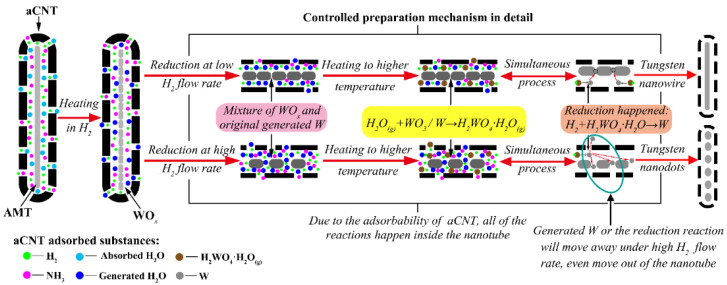
Illustration of the morphology-controllable reduction mechanism inside aCNTs (AMT: ammonium metatungstate), reproduced with permission from Ref. [114], copyright 2020, Elsevier.

Because of their hollow core and tubular structure as well as physicochemical stability, aCNTs have great potential as catalyst supports or templates. For example, aCNT-MoSe_2_ as a support has excellent electrocatalytic hydrogen evolution reaction activity in an acidic medium, whereas an aCNT/ZnS nanocomposite shows photocatalytic activity for dye photodegradation [84,103]. In turn, GaN, Al_2_O_3_, ZnO, and MoO_3_ brushes as templates can be prepared from aCNT brushes [25,93]. Given the capillarity and the unique structure of aCNTs, the latter are used as templates to prepare W nanowires, W nanodots (Figure 15), and tungsten carbide nanowires or nanodots (Figure 16). Furthermore, the special reduction mechanism inside the aCNTs promotes their ability to function as supports or templates (Figure 17) [31,114,128]. To utilize the hollow core of aCNTs, the Marangoni flow was designed and several transition bimetal oxides were deposited onto aCNTs to achieve outstanding electrochemical properties [36,129]. In addition, sharp-edged microflakes and self-assembled microflowers of europium hydroxycarbonate can be prepared from aCNTs [62]. Moreover, aCNTs have been recognized as the proto-form of CNTs [130]. It is worth noting that aCNTs can be unzipped to amorphous graphene under ultrasonic treatment so as to be applied in cold cathodes [78].

### 5.4. Other Applications

In addition to electrical applications and their use as absorbers, supports, and templates, the composites obtained from nanomaterials loaded on or encapsulated into aCNTs can be provided with a certain property or the combined properties can be improved, which enables aCNTs to be applied in a wider range of fields. For example, nanohybrids of aCNT and transition metal dichalcogenides have application potential for many fields, e.g., the easily produced aCNT-MoS_2_ can serve in the removal of toxic dyes from water or as a supercapacitor [63,67,73]. The TiO_2_-aCNT nanohybrid shows improved photocatalytic properties, and the nanohybrid of aligned TiO_2_ nanotubes coated with AaCNTs exhibits a high field emission current density and application potential in the fields of dye or colorant adsorption and of field emission [43,56,57,58]. Combined with MnO_2_ (or with polypyrrole as a ternary nanohybrid), the aCNT-MnO_2_ nanohybrid demonstrates excellent energy storage and field emission properties [42,70,71,72,89,131]. When decorated with CuO, ZnO, ZnS, or copper phthalocyanine, aCNTs show improved field emission and optical properties [75,79,85,91,132]. Furthermore, aCNT-polyaniline core–shell nanostructures can be efficient cold cathode materials, and an aCNT-polypyrrole composite demonstrates enhanced electrocatalytic oxygen reduction activity [74,90].

**Figure 18:**
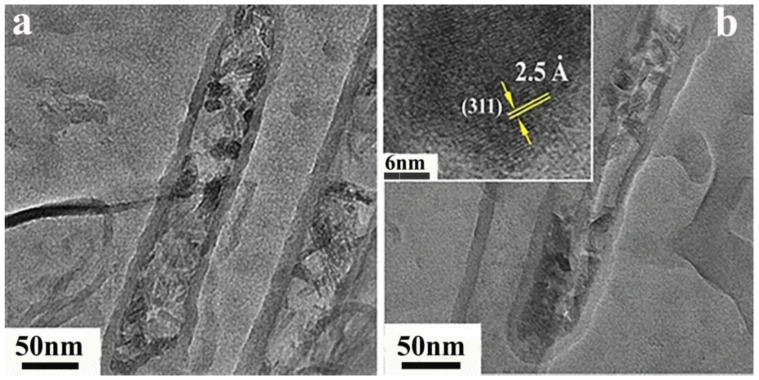
Transmission electron micrographs (**a**,**b**) of aCNTs encapsulated with NZFO, inset of (**b**) is (311) crystallographic planes of NZFO, reproduced with permission from Ref. [61], copyright 2013, Springer.

Interestingly, due to their thermal stability, aCNTs are promising as heat insulation materials, i.e., sol–gel-prepared aCNT-Al_2_O_3_ nanocomposites can be easily dispersed in engine oil and can significantly improve the thermal conductivity of the oil [80]. In combination with MgO, the obtained aCNT composite exhibits a substantially larger effective surface area than the conventional MgO-graphite and significantly less penetration of slag [82]. The heterojunction of an aCNT, Ni nanowire, and MWCNT via end-to-end configuration is characterized by nearly ideal Schottky contacts [133,134]. In addition, aCNTs can be applied as the reinforcement material for polymer (e.g., polyvinyl alcohol) matrices and can improve their thermal, electrical, and mechanical properties [68]. Furthermore, when encapsulated with mixed ferrite (Ni_0.5_Zn_0.5_Fe_2_O_4_, NZFO) via capillary action (Figure 18), the prepared nanohybrid shows variations in low-temperature magnetic behavior and potential as a multifunctional tool [61].

When decorated with SnO_2_, controllably damaged by argon ions on the sidewall, or combined with RGO on carbon cloth/polyethylene terephthalate substrates, aCNT-based nanostructures have good field-emission characteristics [66,83,135]. Furthermore, the amorphous structure of aCNTs makes them a better simulant of kerogen than graphitic CNTs [136].

## 6. Conclusions

Characterized by a straight tubular structure and an amorphous carbon shell, aCNTs are versatile nanomaterials. Compared with graphitic CNTs, cheaper aCNTs have the basic characteristics of ultralow density, physicochemical stability, and thermal stability, which are not affected by chirality but also exhibit no agglomeration-related problems. Nanomaterials consisting of aCNTs can be applied in the fields of environmental protection, catalyst supports, battery electrodes, wave absorption, matrix strengthening, and others. Unfortunately, aCNTs have not yet received as much attention from researchers as graphitic CNTs. This is because the amorphous structure of aCNTs reduces their properties relative to SWCNTs or MWCNTs, even though the characteristics of the former can be improved through combination with other materials. Therefore, this review summarized the preparation methods, structural features, and potential applications of aCNTs to stimulate more investigations on these composites. We believe that the hollow and straight tubular core of aCNTs, as well as their amorphous carbon shell with distributed defects alongside their low cost and large-scale preparation, may enable aCNTs to play a decisive role in a variety of applications, such as nanostructure templates, dye or toxic substance adsorption, catalyst supports, and reinforcements.

## Figures and Tables

**Table 3:** Examples of the application of aCNTs.

The aCNTs’ Characteristics	Application Fields	Examples	Ref.
Structure	Template	Tungsten nanowires	[114]
	Support	Support MoSe_2_ nanosheets used in hybrid hydrogen evolution reaction	[84]
	Reinforcement	Enhancing polymer	[68]
Electrical property	Supercapacitor	Metal-oxide-based supercapacitors, i.e., aCNT manganese di-oxide (MnO_2_)-poly pyrrole (PPy) ternary nanocomposites	[72]
	Battery	LIB anodes	[37]
Adsorption	Pollution	Removal of organic dyes from water	[77]
	Microwave	Electromagnetic wave absorption	[46]
Others	Field emission	Nano-CuO-decorated aCNTs	[75]
	Graphite substitute		[82]

## Data Availability

Not applicable.
